# Dendrosomal nanocurcumin prevents EBV-associated cell transformation by targeting the lytic cycle genes of the Epstein-Barr virus in the generation of lymphoblastoid cell line

**DOI:** 10.22038/IJBMS.2023.69839.15199

**Published:** 2023

**Authors:** Mahboobeh Cheragh, Majid Sadeghizadeh, Mohammad Hassan Pouriayevali, Masoud Parsania

**Affiliations:** 1 Department of Microbiology, Faculty of Advanced Science and Technology, Tehran Medical Sciences, Islamic Azad University, Tehran, Iran; 2 Department of Genetics, Faculty of Biological Sciences, Tarbiat Modares University, Tehran, Iran; 3 Department of Arboviruses and Viral Hemorrhagic Fevers (National Reference Laboratory), Pasteur Institute of Iran, Tehran, Iran; 4 Department of Microbiology, Faculty of Medicine, Tehran Medical Sciences, Islamic Azad University, Tehran, Iran; 5 Medical Genomics Research Center, Tehran Medical Sciences, Islamic Azad University, Tehran, Iran

**Keywords:** Cell transformation, Curcumin, Epstein-Barr virus, Lymphoma, Viral genes

## Abstract

**Objective(s):**

Targeting the lytic cycle of the Epstein-Barr virus (EBV) has been considered a new treatment strategy for malignancies caused by this virus. This study aimed to investigate the effect of Dendrosomal NanoCurcumin (DNC) to prevent cell transformation and inhibit the expression of viral lytic gene expression in the generation of lymphoblastoid cell line (LCL).

**Materials and Methods:**

Cell viability of LCLs and PBMCs was performed by MTT assay, and flow cytometry (Annexin/PI) was used for evaluation of apoptosis. CD markers on the surface of generated LCL (CD19) cells were examined for cell validation. The effect of DNC on transformation was evaluated by examining cell morphology and determining the expression level of lytic genes BZLF1, Zta, BHRF1, and BRLF1 of EBV using Real-time PCR. Student’s t-test was used for statistical analysis.

**Results:**

The MTT assay showed that DNC can inhibit the proliferation of LCL in a dose-dependent manner. The 50% cytotoxic concentration (CC_50_) of DNC and curcumin for LCL was determined 38.8 µg/ml and 75 µg/ml, respectively after 72 hr. Also, Real-time PCR data analysis showed that DNC in 30 µg/ml concentration significantly inhibited cell transformation in the LCL and significantly reduced viral lytic genes such as BZLF1, Zta, BHRF1, and BRLF1expression compared to control.

**Conclusion:**

Overall, these findings show that DNC reduces the expression of the viral lytic cycle genes and also the induction of cell apoptosis and finally prevents the generation of LCL.

## Introduction

The Epstein-Barr virus (EBV) is a carcinogenic virus that is directly related to potentially infecting B lymphocytes and pharyngeal epithelial cells (1, 2). EBV is a member of the *Herpesviridae* family belonging to the *gammaherpesvirinae* subfamily of the *Lymphocryptovirus* (LCV) genus (3). Given that 95% of the global population is infected with EBV, it shows the importance of this virus in the world. This virus mainly causes infectious mononucleosis and is closely related to both lymphatic and epithelial malignancies such as Burkett’s lymphoma (BL), Hodgkin’s lymphoma (HL), Nasopharyngeal carcinoma (NPC), AIDS-associated B cell lymphoma and several types of lymphoproliferative diseases (LPDs) (4).

EBV, can cause two modes of latency and lytic pathway in its host cell and express viral latent or lytic genes for induction of the tumor cells in lymphoproliferative disease associated with EBV (5). The virus can express four different types of latency patterns, such as type I, II, III, and W promotor (Wp) (6). This virus can express different genes of the latent cycle; type III latency is detected in AIDS-associated B cell lymphoma, post-transplant lymphoproliferative disorders (PTLD), and lymphoblastoid cell line (LCL), *in vitro* model of EBV-LPDs. Under certain conditions, the latent virus type III changes from its latent stage to the lytic phase which eventually leads to new infectious particles (7). Many EBV-related diseases occur in a latent stage but studies in recent years have shown that genes expressed in the lytic cycle of the EBV virus are extremely important in the carcinogenesis of various types of cancer (8, 9).

In the lytic phase, the expression of EBV genes during replication occurs in 3 stages, immediate-early (IE), early (E), and late (L). Expression of precursor genes such as BamHI Z fragment leftward open reading frame 1 (BZLF1), and Bam HI fragment R rightward open reading frame1 (BRLF1) (IE- immediate early) have been shown to induce the TNF receptor-associated factor c-Jun N-terminal kinases (TRAF2-JNK) pathway and lytic replication of the virus (10). These genes can activate promoters of lytic E genes. Then the lytic L genes encode structural proteins and afterward viral packaging is expressed. On the other hand, researchers have shown that the Z- transcriptional activator protein (Zta) acts on activating immunosuppressive cytokines such as interleukin-10 (IL-10) and interleukin-13 (IL-13). These cytokines can play a very important role in tumor growth through the activation of vascular endothelial growth factor (VEGF) protein (11, 12). Furthermore, Bam HI fragment H rightward open reading frame 1 (BHRF1), one of the early proteins of the lytic cycle that is homologous to human Bcl2 anti-apoptotic protein, both in terms of genomic sequence and function, is able to inhibit apoptosis in EBV-infected cancer cells (13, 14). Transformation of normal B lymphocytes by secreted EBV within the supernatant of B 95-8 cell culture (Lymphoblastoid Marmoset Cell Line) is a suitable model system ​for investigating the B cell transformation and viral lytic reactivation (15-17).

In recent years, the tendency to find drugs or compounds of natural origin with fewer side effects than chemotherapy and chemotherapeutic drugs has increased dramatically. The natural composition of curcumin is a good candidate for cancer research due to its multi-purpose nature and lack of side effects on normal cells (18). In general, the most important biological effects of curcumin are its anti-inflammatory, anti-tumor, and anti-oxidant effects. During several studies on curcumin, it was found that this compound induces apoptosis by inhibiting various molecular targets and inhibits metastasis, invasion, and angiogenesis (19-21). However, despite the excellent therapeutic properties of curcumin, the compound has been shown to have low bioavailability due to its low intestinal absorption (22). Therefore, new strategies to increase the bioavailability of this compound are being studied and one of them is to create the nanoparticle of curcumin by dendrosome (23-28).

Nanotechnology is a good opportunity to produce small-scale delivery systems called nanocarriers that increase the bioavailability of substances such as curcumin in the body. Dendrosome is an amphipathic biodegradable nanocarrier combined with curcumin by Dr. Sadeghizadeh’s research group and synthesized as DNC at Tarbiat Modares University and provided to our group. This nanocarrier has been demonstrated to present enormous efficacy as a drug delivery platform in various research *in vitro* and *in vivo *(27, 29-32). It has also been shown in previous research that the effective doses of dendrosomal nanocarrier for anticancer drug delivery do not exert any toxic effects on normal human cells (33-35).

This study aimed to investigate the potential effect of DNC inhibiting cell transformation and EBV lytic cycle gene expression.

## Materials and Methods

***Cell lines and virus***

The marmoset lymphoblastic cell line B95-8 was purchased from the Iranian biological resource center. This cell line was used as the potential source of high titers of EBV particles for the transformation of PBMCs to the LCL. The cell line was grown in RPMI1640 (Gibco) supplemented with 10% fetal bovine serum (FBS) (Gibco), 100 IU/ml penicillin, and 100 μg/ml streptomycin (Gibco, UK) and cultured in 75 cm² flasks using a sterile technique and incubated at 37 °C in a 5% CO_2_. The supernatant was separated and after centrifugation at 400×g for 10 min, the solution was filtered by passing through the 0.22 µm filter (36).

***Cell transformation and generation of LCL***

The peripheral blood mononuclear cells (PBMCs) as normal cells were isolated from the whole blood of a healthy person according to the previously optimized protocol. Briefly, whole blood with ethylenediaminetetraacetic acid (EDTA) as anticoagulant was collected from a healthy person, and mononuclear cells were separated with Ficoll-lymphodex (inno-train). After centrifugation, the buffy coat was removed and the cells were washed twice with PBS. The amount of 0.04 mg/ml cyclosporine A (Sandimmun –NOVARTIS-50 mg/ml) was added to the medium for inhibition growth of T-cells. The cells were added to a 75 cm² tissue culture flask containing RPMI 1640 with 10% FBS and were incubated at 37 °C in a 5% CO_2_.

After that, 500 µl of filtered supernatant of B95-8 cell line, containing EBV was adjacent to 2× 10⁶/ml normal cells and 4 ml transfer medium including 0.04 mg/ml cyclosporine A, 10% FBS, and 20 μg/ml phytohemagglutinin (Gibco) as a mitogen was added to a 25 cm² tissue culture flask and was incubated at 37 °C in a 5% CO_2_. The formation of rosette-like transformed LCLs was observed under the microscope after two weeks (37-39).

***Confirmation of cell transformation by evaluating surface CD markers***

After transforming normal lymphocytes into lymphoblastoid cells, they were examined under a microscope for rosette-like morphology, and after determining viability up to 85–90%, CD3 as a surface marker of T cells and CD19 as a surface marker of B cells were examined by flow cytometry analysis (40).

***Cellular cytotoxicity test***

The MTT (3-(4,5-dimethylthiazol-2-yl)-2,5-diphenyl-2H-tetrazolium bromide) method was used to determine the cytotoxicity of curcumin, dendrosome, and DNC on LCL and the normal cells as a control. Briefly, LCL and normal cells were seeded separately onto 96-well plates with the amount of 30×10³ cells/well in triplicates for 72 hr. Afterward, cells were treated with different concentrations of DNC (0,10,20,30,40,50,60 μg/ml), curcumin (0,10,20,30,40,50,60,70,80,90, and100 μg/ml), and dendrosome (0,600,800,1000, and 1200 μg/ml). After incubating for 72 hr the medium was removed, 20 μl of MTT reagent (5 mg/ml in PBS) was added to each well, then 80 μl RPMI was added without FBS to each well, and was incubated at 37 °C for 4 hr, then 100 μl of dimethyl sulfoxide (DMSO) solution was added to wells. After a 10-minute incubation in a dark place, the optical density of the wells was measured at 540 nm using a microliter plate reader (Stat Fax 3200 Microplate Reader, USA). All values were compared to the corresponding controls (untreated cells). Cell viability was calculated as the ratio of the absorbance of treated cells to untreated control cells. The following formula was used for determining the percentage of viable cells in the MTT assay. Where: Optical density (OD) t is the mean OD 540 nm value from treated cells, and OD u is the mean OD 540 nm value derived from untreated control cells (41, 42).

$\mathrm{Viable cells}\left(\%\right)=\frac{\mathrm{OD t}(\mathrm{average of the absorbance value of treated cells})}{\mathrm{OD u}(\mathrm{average of absorbance value of the corresponding untreated cells}}\times 100$

The effect of DNC, dendrosome, and curcumin separately on LCL and normal lymphocytes was examined triplicated in 72 hr.

***Apoptosis analysis by flow cytometry***

Flow cytometry technique with FITC-conjugated Annexin V and propidium iodide (PI) was used to investigate cell apoptosis. LCL and normal cells were treated with four concentrations of DNC (0, 10, 20, 30, and 40 μg/ml) and incubated for 72 hr at 37 °C, 5% CO2. After 72 hr the cells were evaluated by flow cytometry (BD FACS Calibur; Becton-Dickinson, USA), using an Annexin V apoptosis detection Kit (IQ products, Netherlands) according to the standard protocol of the manufacturer. Data analysis was done by FlowJo software (version 10, USA).

***RNA extraction and reverse transcription PCR***

Total cellular RNA was extracted from LCL after 72 hr treatment with four concentrations of DNC (0, 10, 20, 30, and 40 μg/ml) using the Favorgen (Biotech Corp) kit according to the kit’s standard protocol. Single-stranded complementary DNA (cDNA) was synthesized using a cDNA reverse transcription kit (Yektatajhiz Azma) following the manufacturer’s protocol.

***Quantitative Real-time PCR***

Evaluation of BZLF1, Zta, BHRF, and BRLF gene expression was performed by a relative quantification method using Real-time PCR, and GAPDH was used as a reference gene. The primer sequences of BZLF, Zta, BHRF, BRLF, and GAPDH genes were shown in Table 1. The final volume in Real-time PCR reaction was 20 μl with 2 ng of cDNA template, 10 μl SYBER Green master mix (Yektatajhiz Azma), and 0.5 μl of forward and reverse primers. Amplification for all primers was performed following initial denaturation at 95 °C for 600 sec, followed by 40 cycles of denaturation at 95 °C for 25 sec, annealing at 60 °C for 25 sec, and extension at 72 °C for 25 sec. Quantitative PCR was performed using Light Cycler® 96 Instrument (Roche Molecular Systems, Germany). The relative expression for each gene was carried out using the ΔΔCt method. 

***Statistical analysis***

Student t-test was used for statistical and significance analysis level calculation between the two groups, and an ANOVA test was used for more than two groups. Differences in gene expression were determined using Genex 6 software, statistical analysis by using SPSS 21 software, and charts were made with graph pad prism 8. A *P*-value less than 0.05 was considered significant.

## Results

***Lymphocyte Transformation***

**Figure 1:**
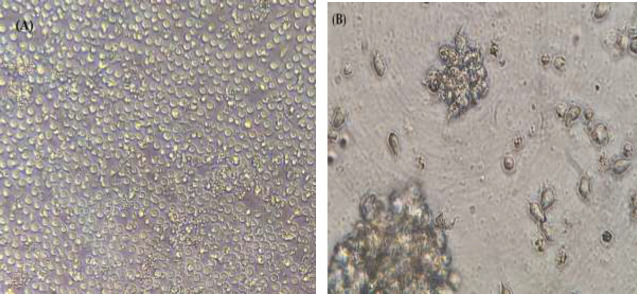
Morphology of LCL before and after transformation

The transformation of PBMCs was evaluated by examining the changes in cell morphology; including the formation of cell masses, increasing the size of cells, and rosette-like shapes (Figure 1). At this stage, LCLs were cultured in a 25 cm^2^ cell culture flask with an RPMI 1640 culture medium containing 0.04 mg/ml cyclosporine A and 10% FBS, for further evaluation.

***Evaluation of CD markers***

**Figure 2.**
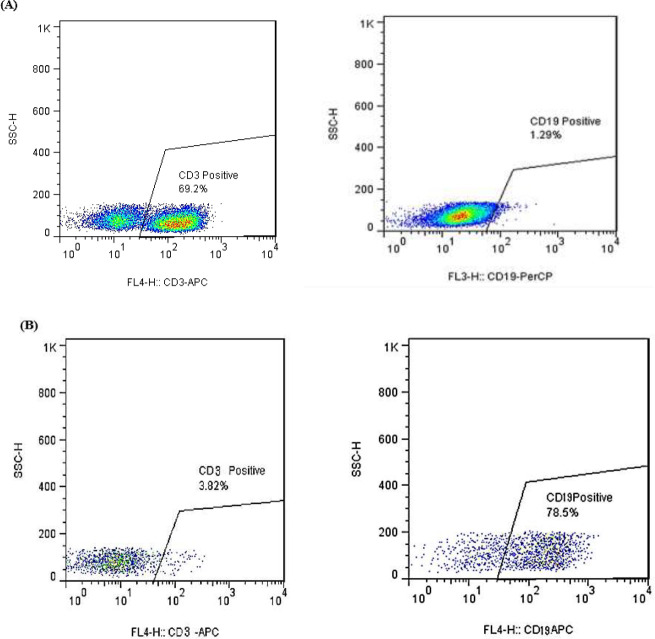
Lymphoblastoid cells surface marker

Surface markers of LCL after three times of cell subculture (Figure 2) were analyzed by flow cytometry, normal cells were considered as control and showed a high level of CD3 (69.2%) and low values of CD19 (1.29%) (Figure 2A). LCL showed a low level of CD3 marker (3.82%) and a high level of CD19 (78.5%) (Figure 2B).

***Cellular cytotoxicity***

**Figure 3:**
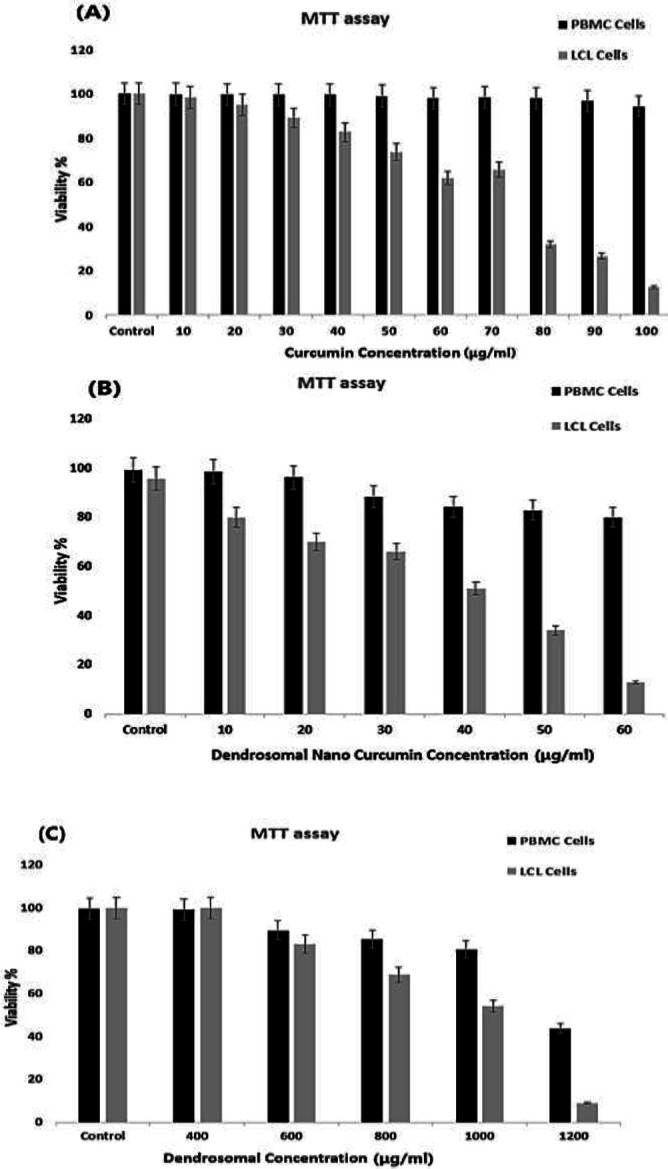
MTT assay to assess cell viability

As shown in Figure 3, the 50% cytotoxic concentration (CC_50_) of curcumin, DNC, and dendrosome on LCL were determined at 75 μg/ml for curcumin, 38.8 μg/ml for DNC, 1000 μg/ml for dendrosome, and these values for PBMCs were assessed at 100 μg/ml for curcumin, at 90 μg/ml for DNC, and 1200 μg/ml for dendrosome after 72 hr, using the MTT assay. After comparing DNC with curcumin, we used DNC due to its greater effectiveness and fewer side effects in the rest of the study.

***DNC effect on apoptosis in LCL and normal cells***

**Figure 4:**
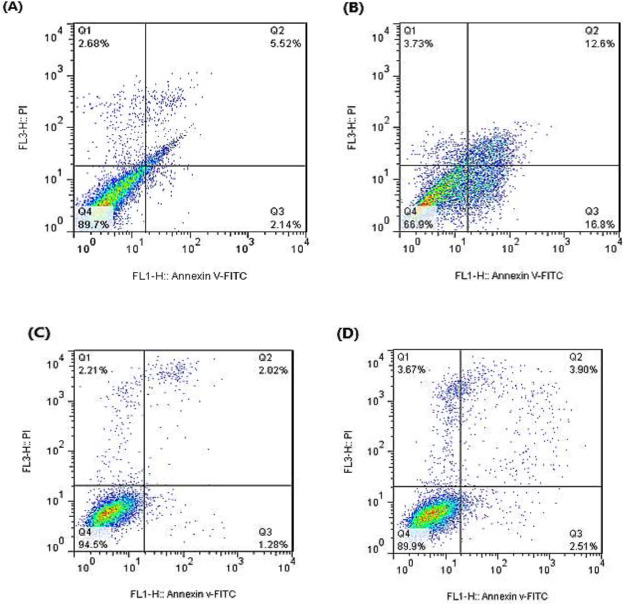
Apoptosis results of DNC effect on LCL compared to PBMCs by flow cytometry

The cell apoptosis effect of DNC on the LCL and PBMCs treated with CC_50_ of DNC were examined by flow cytometry. As shown in Figure 4, DNC has an inhibitory effect on cancer cells, but at the same concentration has no adverse impact on normal cells. The results of cell apoptosis by Annexin V/PI showed that the CC_50_ of DNC on the LCL caused 16.8% early apoptosis and 12.6% late apoptosis, and on PBMCs caused 2.51% early apoptosis and 3.90% late apoptosis.

***Inhibitory effect of DNC on transformation***

**Figure 5:**
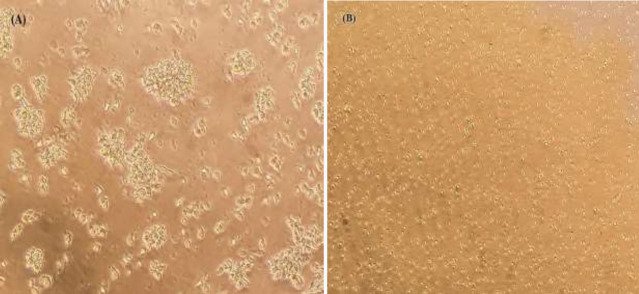
Microscopic image of PBMCS and LCL for demonstrating inhibitory effect of DNC on cell transformation by cluster formation

As shown in Figure 5, PBMCs that were not treated with DNC were considered as control and cell transformation occurred on them, but PBMCs were treated with a culture medium containing 30 μg/ml of DNC (subtoxic dose of DNC) due to the inhibitory effect of DNC, cell transformation did not occur and cell clusters were not formed in PBMCs.

***Gene expression by Real-time quantitative PCR***

**Figure 6:**
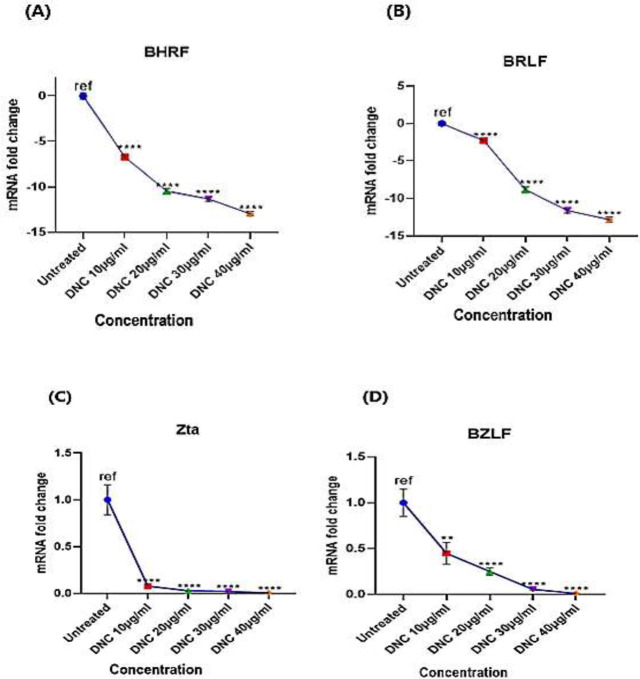
Gene expression of BHRF1, BRLF1, BZLF1, and Zta in LCL cells after 72 hr treatment with 10, 20, 30, and 40 μg/ml of DNC. (A and B) Results of BHRF1 and BRLF1 gene expression in LCL show all four doses of DNC cause a significant decrease in gene expression compared to the control. (C) Expression of the Zta gene shows a significant decrease in all four concentrations of DNC compared to the control group. (D) There is a significant variation of BZLF1 gene expression at 10 μg/ml of DNC treatment and 20, 30, and 40 μg/ml concentrations. The average fold change value of 3 independent experiments as demonstrated in the graph. ** *P*-value= 0.001–0.01, ****P-value˂0.0001

The expression of viral genes after treating LCLs with different concentrations of DNC (0, 10, 20, 30, and 40 μg/ml) was evaluated by quantitative Real-time PCR. The expression of BHRF1, BRLF1, and Zta genes at 10 μg/ml of DNC significantly decreased compared to the control group (*P*<0.0001) (Figure 6 A, B, D). As shown in Figure 6C, BZLF1 gene expression at 10 μg/ml of DNC had a significant decrease compared to the control group (*P*>0.01). In general, the results show that the expression of BHRF1, BRLF1, BZLF1, and Zta genes decreased significantly in a dose-dependent manner compared to the control group (*P*<0.0001).

**Table 1:** Characterization of oligonucleotide primers used for Real-time PCR

**Gene**	**Primer sequence (5** **′** **→3** **′** **)**	**product length (bp)**
Zta	F CTATCAGGACCTGGGAGGGC R CACAGCACACAAGGCAAAGG	51
BHRF1	F GTACCCTGCATCCTGTGTTG R CTACAGTGTCCTCTGGCGAA	72
BRLF1	F ATGGAACATGCGTCGTTG R AATGGCCACGCTCAACAT	96
BZLF1	F AAATTTAAGAGATCCTCGTGTAAAACATC R CGCCTCCTGTTGAAGCAGAT	92
GAPDH	F CTCTTGCTACTCTGCTCTG R GCCTGCCTGGTGATAATC	179

Zta: Z- transcriptional activator protein; BHRF1: Bam HI fragment H rightward open reading frame 1; BRLF1: Bam HI fragment R rightward open reading frame1; BZLF1: BamHI Z fragment leftward open reading frame 1; GAPDH: Glyceraldehyde-3-phosphate dehydrogenase; bp: base pair; F: Forword; R: Reverse

(A)PBMCs before transformation (with magnification 20X); (B) enlarged cells with cluster and rosette-like shape of LCL after transformation (with magnification 20X)

LCL: Lymphoblastoid cell line; PBMC: Peripheral blood mononuclear cells

(A) Normal cells as a control group showed a high level of Tcell marker (CD3) and low level of B Cell-specific marker (CD19) ;(B) LCLs show a high level of B Cell-specific marker (CD19) and low level of Tcell marker (CD3)

LCL: Lymphoblastoid cell line; PBMC: Peripheral blood mononuclear cells; CD: cluster of differentiation

Effect of curcumin, DNC, and dendrosome on cytotoxicity of LCL and PBMCs at 72 hr. (A) Effect of curcumin on LCL and PBMCs, (B) Effect of DNC on LCL and PBMCs, and (C) Effect of Dendrosome on LCL and PBMCs. The average of three independent experiments is shown in the graph. Error bars indicate standard deviation

MTT: 3-(4,5-dimethylthiazol-2-yl)-2,5-diphenyl-2H-tetrazolium bromide; LCL: Lymphoblastoid cell line; PBMC: Peripheral blood mononuclear cells; DNC: Dendrosomal nanocurcumin

(A) Control LCL, (B) 30 μg/ml concentration of DNC on LCL, (C) Control PBMC, (D) 30 μg/ml concentration of DNC on PBMC

(A) PBMCs treated with culture medium without DNC and formation of cell clusters that show signs of cell transformation after 72 hr (10X magnification); (B) PBMCs treated with culture medium containing 30 μg/ml DNC showed no signs of cell clusters after 72 hr (10X magnification)

LCL: Lymphoblastoid cell line; PBMC: Peripheral blood mononuclear cells; DNC: Dendrosomal nanocurcumin

Zta: Z- transcriptional activator protein; BHRF1: Bam HI fragment H rightward open reading frame 1; BRLF1: Bam HI fragment R rightward open reading frame1; BZLF1: BamHI Z fragment leftward open reading frame 1.

## Discussion

In the present study, due to the importance of cell transformation in EBV-associated malignancies, the effect of DNC as a natural substance on PBMC, after EBV is released by B95-8 cells, was investigated. As mentioned, the results of this research showed that DNC as a natural substance reduces the expression of the viral lytic cycle genes and has the potential to induce apoptosis and prevents the generation of LCL. The EBV specifically infects B cells and activates them. Lymphoblastoid cells are the result of the transformation of normal lymphocytes by EBV. Since EBV-infected B lymphocytes have a high content of lytic genes, they are suitable model cells for studies related to the role of EBV in malignancy (43, 44). The EBV lytic cycle expresses a set of genes such as BHRF1, BZLF1, Zta, and BRLF1 which play an important role in the replication of the virus and inducing cell transformation (45, 46).

In order to initially activate the lytic phase of the virus, the expression of two transcription factors BZLF1 and BRLF1 is required for the replication of EBV genomic DNA (47). The expression of the BZLF1 gene leads to the expression of Zta, which induces the expression of some interleukins such as interleukin-10 (IL-10) as well as interleukin-13 (IL-13) (48, 49). These cytokines cause cellular VEGF secretion, which induces tumor growth (50).

In 2017, Stanfield *et al*. showed that BZLF1 and BRLF1 genes play an important role in cell carcinogenesis by investigating and studying the biological and structural function of latency genes (EBNA1, 2, 3) and NF-kBoMyc signaling pathways in the lytic virus cycle. These genes have been investigated in the laboratory mouse model of different cell types (51). In the present study, we showed that the expression of BZLF1 and BRLF1 genes is significantly reduced in cells treated with DNC, and this can lead to a reduction in the replication of the virus genome and exert its inhibitory effect on the replication of the virus.

The expression of the Zta gene leads to reduction of TNF-R1 and inhibition of TNF-α, which is an important factor in the induction of apoptosis in cancer cells (52). In this research, we have shown that the expression of Zta in cells treated with 30 μg/ml of DNC significantly decreases. By reducing the expression of this gene, the process of apoptosis in the cell infected with the virus is strengthened through the above genes.

BHRF1 leads to increased anti-apoptotic gene expression such as of bcl2 which may cause genomic instability and progress into cancer cells (53). In this study, we have shown that a decrease in BHRF1 can lead to increased apoptosis.

In 2018, Li *et al*. studied the lytic cycle of EBV and its relationship with carcinogenesis. In addition, they investigated the inhibition of the lytic cycle by two groups of natural polyphenolic compounds such as resveratrol and sulforaphane, and flavonoids such as genistein and luteolin. The lytic cycle of the virus has been effective in preventing excessive cell proliferation through inhibition and down-regulation of BZLF1(Zta), BRLF1(Rta), and BHRF1(homolog bcl2) genes (54).

During this study, we also showed that cell transformation occurred in the cells that were not treated with DNC following EBV infection, but the EBV-infected cells that were treated with 30 μg/ml of DNC did not undergo cell transformation due to the significant reduction of lytic gene expressions such as BHRF1, BZLF1, Zta, and BRLF1, and this can indicate an important role of curcumin in the prevention of cell transformation, which is consistent with the Li study. One of the important pieces of evidence in the process of successful transformation of PBMCs to LCL by the EBV is the decrease in the number of T lymphocytes due to the inhibitory effect of cyclosporine A. Other evidence of this phenomenon is the strengthening of the proliferation of B lymphocytes due to EBV infection. In the present study, after examining the surface markers of PBMCs and comparing them with LCL, it was shown that PBMCs have a high level of CD3 (T-cell marker) and a low level of CD19 (B-cell marker), while LCL showed a low level of CD3 and a higher level of CD19; a high level of CD19 in LCL cells indicates that B cells have been infected and immortalized by EBV.

The investigation of CD markers on the surface of B cells has been done by other researchers such as Yap and colleagues. This group realized that CD19 has a significant increase in the surface of LCL compared to PBMCs, after examining the surface markers of PBMCs (55).

In another study conducted by Hur and colleagues, they showed that LCLs were confirmed by transfection of CD19 and B cell receptors (BCR). During their research in 2005, this group showed that the surface marker of B cells has been confirmed with high levels of CD19 and low levels of CD3 (56).

Curcumin is one of the natural compounds with many therapeutic properties that has attracted researchers due to its multipurpose therapeutic nature and its effect on cancer and antiviral activity (57). In 2021, Šudomová and Sherif demonstrated the antiviral effect of curcumin on human and animal herpes viruses; they investigated the mechanisms and pathways of curcumin effects on its targets and confirmed that this substance can destroy both latent and lytic phases of the virus (58).

In 2018, Liu and colleagues investigated the effect of curcumin on the proliferation, cycle arrest, and apoptosis of Epstein-Barr-associated human nasopharyngeal carcinoma Cells (NPC). This group showed that curcumin inhibited EBV latent and lytic cycle replication in both HONE1 and HK1-EBV cells. Curcumin can down-regulate EBNA1 expression and exert antitumor therapy of NPC *in vitro *(59).

In general, from the results obtained in this study, it can be concluded that DNC is able to induce apoptosis of Epstein-Barr-associated cancer cells by targeting the genes of the lytic phase cycle and inhibiting the expression of the important genes of the lytic cycle of the virus. In addition to being a medicinal compound extracted from a natural plant, this compound can be effective in preventing and controlling cancers caused by EBV. Finally, additional studies on other lytic phase genes of EBV *in vivo* and *in vitro* environments are suggested.

## Conclusion

According to the results obtained in this study, DNC can affect the transformation of PBMCs and direct them toward cell apoptosis, in addition, this compound can inhibit the expression of Epstein virus lytic phase genes. Anti-EBV combinations, especially natural ones such as curcumin with nanomaterial such as dendrosome, could be adequate candidates for the prevention and treatment of cancers caused by EBV. 

## Authors’ Contributions

M P and M S conceived and designed this study. M P and M CH contributed to the writing of this manuscript, finding references, and laboratory experiments. M S carried out the design and construction of DNC and provided necessary materials such as curcumin and dendrosomes for the study. M P, MH P, and M CH analyzed gene expression data. M S and M P edited and finalized the manuscript. All authors read and approved the final manuscript.

## Funding

Not applicable.

## Data Availability Statement

All experiments and data analysis of this research is published in this manuscript.

## Ethis Approval and Consent to Participate

This study has an ethical approval (code number IR.IAU.PS.REC.1398.235) from Tehran Islamic Azad Medical Sciences University.

## Conflicts of Interest

The authors declare that there are no conflicts of interest.
